# Family criminal legal system exposure and early adolescents’ pubertal development: the mediating role of family strain

**DOI:** 10.1093/aje/kwae457

**Published:** 2024-12-13

**Authors:** Juan Del Toro, Michael Roettger, Dylan B Jackson, Sylia Wilson

**Affiliations:** Department of Psychology, University of Minnesota-Twin Cities, Minneapolis, MN 55455, United States; School of Demography, Australian National University, Canberra, ACT, Australia; Department of Population, Family and Reproductive Health, Johns Hopkins Bloomberg School of Public Health, Baltimore, MD, United States; Institute of Child Development, University of Minnesota-Twin Cities, Minneapolis, MN 55455, United States

**Keywords:** criminal legal system, pubertal development, adolescence

## Abstract

Pubertal trends, wherein adolescents today are experiencing puberty earlier than prior generations, have coincided with the expansion of the criminal legal system, which is disproportionately affecting communities of color. However, whether pubertal development and criminal legal system exposure among adolescents are interrelated is unknown. We tested whether family members’ criminal legal system exposure predicted adolescents’ pubertal development, whether family strain explained the relation between criminal legal system exposure and pubertal development, and whether race/ethnicity moderated our results. We used 3 yearly waves of longitudinal data among a national sample of 9518 adolescents. Results indicated that 40% of Black, 20% of Latinx, 16% of adolescents of other races or ethnicities, and 10% of White adolescents experienced 1 or more family criminal legal system exposures. In structural equation models within a case-crossover design controlling for measured confounders and unmeasured confounders that do not change over time, including neighborhood-level socioeconomic status and crime, family criminal legal system exposure predicted adolescents’ advanced pubertal development, and family strain explained this relation between family criminal legal system exposure and pubertal development. The approach to law and order in the United States has public health implications that may be perpetuating health inequities, as accelerated pubertal development can have downstream consequences across the life course.

## Introduction

Since the 1970s, the “War on Drugs” and “War on Crime” in the United States has led to mass imprisonment and criminal justice involvement, exposing tens of millions of children to the criminal legal system over multiple generations.^1-3^ One in 10 American adults serves time in jail or prison in their lifetime, which is the highest rate of incarcerated adults in the world, and this rate is 1 in 3 among Black Americans.^4^ Many of these incarcerated adults have family members and children in their households, positioning children as vicariously experiencing family members’ criminal legal system exposure. Indeed, 6% of a nationally representative sample of children under the age of 18 years have experienced a parent undergo imprisonment,^5^ with a higher prevalence of 21% among children in low-income communities of color.^6^ By adulthood, 35% of young adults aged 18 to 29 years have had a parent incarcerated among a nationally representative sample of adults surveyed in 2018.^7^ However, parental incarceration is 1 aspect of and is among the most commonly studied children’s family criminal legal system exposure.^8^ Here, we provide a multidimensional picture of family criminal legal system exposure that includes family members’ arrests and other forms of contact with criminal legal authorities.

Family criminal legal system exposure is common among adolescents of color.^9^ Because of intersecting racial/ethnic and gendered stereotypes that portray young men of color as more troubling and delinquent than their White counterparts, men of color are disproportionately more likely to be taken into custody and receive lengthier and harsher sentences than White individuals in the United States.^10^^,^^11^ Therefore, youth of color are more likely to have male family members, such as fathers, be stopped by the police and experience incarceration.^6^^,^^12^ In addition, families of color have fewer resources, such as paying bail or obtaining adequate legal counsel, to cope with a family member’s arrest and prosecution.^9^ These resources help prevent legal-system-exposed family members from being incarcerated as pretrial detainees.^9^ These limited resources stem from historical prejudicial practices (eg, redlining)^13^ that concentrated socioeconomic disadvantage among communities of color. Therefore, not only are youth of color more likely to experience family criminal legal system exposure but the severity and stigma associated with such exposures may be more detrimental to their development.^14^

Family criminal legal system exposure can have cascading consequences on adolescent development via family strain. The ambiguity and repercussions of incarceration (eg, job loss, financial instability, loss of health insurance) can lead mothers of children to view incarcerated fathers as unsuitable intimate or marriageable partners.^15-17^ Possible parental separation during a youth’s early years can be traumatizing and present economic challenges during their upbringing.^18^ Even less severe vicarious contact with the legal system (eg, police stops and arrests) can trigger adolescents’ shared-fate visions and anticipation of unfair treatment from the legal system.^14^^,^^19^^,^^20^ Thus, family members’ negative exposure to the criminal legal system may influence the household climate (eg, parental job loss and separation) and, in turn, their psychosocial,^8^^,^^21^ academic,^22^^,^^23^ and physiological maladjustment.^6^^,^^24^^,^^25^ However, prior studies relied on unique samples (eg, single-parent households^6^ or households with an incarcerated parent),^25^ limiting their generalizability, because it can be unclear whether extant findings are attributable to criminal legal system exposure or to unobserved time-invariant confounders (eg, crime, socioeconomic disadvantage). With a relatively more representative sample of children in the United States, we expand our knowledge about the consequences linked to the criminal legal system.

One specific facet of adolescent development that has lasting implications over the life course is pubertal timing.^26^ Puberty represents the onset of adolescence, and more advanced pubertal development indicates accelerated physiological maturation and aging.^26^^,^^27^ Early puberty can have social consequences and lead individuals to view and treat more physically matured adolescents as older, less innocent, and more menacing, even when youth do not engage in any delinquent behaviors.^28^ Adverse childhood experiences (ACEs) have predicted physical maturation via advanced pubertal development^29^ and more accelerated cellular aging.^6^^,^^30-33^ Stressful events linked to parental incarceration have also predicted physiological indicators of stress (eg, cortisol secretion),^25^ inflammation,^34^ and allostatic load.^35^^,^^36^ Moreover, puberty often coincides with the onset of antisocial behaviors and conduct disorders, which may account for the intergenerational transmission of offending and imprisonment^37-39^ and concentrate social and health disadvantages among communities of color. For instance, in a large study of adolescents, youth with incarcerated parents were more likely to experience 4 or more ACEs and more mental health services, but Black and Latinx youth with incarcerated parents were less likely to receive mental health services than were White youth with incarcerated parents.^40^

These external factors, partly stemming from the criminal legal system, may be contributing to an ongoing crisis affecting most adolescents of color: the adolescent mental health crisis.^41^^,^^42^ This crisis is partly attributable to chronic stressors leading youth to experience pubertal development earlier than prior generations, because advanced pubertal development (before the development of advanced self-regulatory skills) leaves youth acutely sensitive to adversities in their environments,^43^^,^^44^ including those that may be racially or ethnically motivated (eg, discrimination). As the data show in a US survey of 200 000 youth aged 12 to 17 years, rates of major depressive episodes increased by 52% between 2005 and 2017.^45^ During this period, suicide rates increased by 106% for Black youth, 82% for Latinx youth, but by 39% for White youth,^46^ suggesting that racial/ethnic disparities in mental health are not entirely attributable to disparities in diagnoses. Therefore, our assessment of environmental factors can inform targeted interventions working to reduce threats to adolescents’ social ecologies.

In the present study, we addressed the following 3 research questions: Do youth with family criminal legal system exposure exhibit more advanced pubertal development? Does family strain mediate the longitudinal inter-relations between family criminal legal system exposure and youth’s pubertal development? And do the rates of and consequences linked to family criminal legal system exposure vary across racial/ethnic groups? Because the criminal legal system reflects an institution that is external to adolescents’ homes, we theorized that family criminal legal system exposure is an independent yet related type of ACE that can lead to more ACEs, including family strain. We also predicted that family criminal legal system exposure would be more prevalent and have worse consequences for adolescents of color than for White adolescents. To test our theories, we shied away from testing general associations among our constructs, because traditional between-person comparisons can yield biased estimates due to measured and unmeasured time-invariant confounders. Instead, we leveraged a case-crossover design, in which participants served as their own controls. This focus on intraindividual change enabled us to make within-person comparisons and compare exposures over time within the same person, conditioning on all stable characteristics of the individual.

## Methods

Data came from 9518 early adolescents who participated in the Adolescent Brain Cognitive Development (ABCD) Study, which is an ongoing longitudinal study of early adolescents (baseline mean age, 9-10 years) across 21 sites in the United States. The ABCD Study encompasses national and yearly longitudinal assessments of pubertal development during pre- and early adolescence, permitting an assessment of potential contributors to youths’ early onset of puberty relative to same-age and -sex peers.

Table 1 presents demographic characteristics for the analytic sample of 9518 adolescents (see Appendix S1 for exclusionary criteria from the initial sample of 11 868 adolescents). The sample as an aggregate reflected a privileged group of youth: 75% of them came from 2-parent households, in which both parents, on average, attained a postsecondary (eg, associate) degree. Table 1 also lists demographic characteristics among the analytic sample comprising 5413 White, 1160 Black, 1734 Latinx, and 1211 other race/ethnicity (hereafter, “Other”) adolescents. After we used the Benjamini-Hochberg false discovery rate method to account for multiple testing,^47^ no significant racial/ethnic group differences emerged by age and sex of the youth, but the overall pattern of the youths’ demographic information suggests those of color were more likely to live in single-parent households, have parents with lower educational degrees and income, and live in census tracts with a greater proportion of residents living below the poverty line and are more racially/ethnically diverse. Notably, Black youth were not more likely than their White peers to live in census tracts where adults engaged in a total number of offenses, but these rates were higher among Latinx and Other youth.

**Table 1:** Demographic characteristics of full analytic sample and sample by racial/ethnic group.

**Key study variable**	**Full sample (*N* = 9518)**	**White youth (*n* = 5413)**	**Black youth (*n* = 1160)**	**Latinx youth (*n* = 1734)**	**Other youth (*n* = 1211)**
Male (vs female) youth, %	52.40	53.10	50.30	52.50	51.40
Youth age at baseline, mean (SD)	9.48 (0.51)	9.49 (0.50)	9.49 (0.51)	9.45 (0.50)	9.48 (0.51)
2 (vs single) parent house	76.30	85.80	38.80	71.70	76.50
Parental education, mean (SD)	16.68 (2.50)	17.47 (1.81)	15.20 (2.40)	14.96 (3.21)	17.02 (2.28)
Parental income, mean (SD)	7.38 (2.32)	8.20 (1.64)	5.07 (2.64)	6.32 (2.39)	7.44 (2.36)
Census tract below poverty, mean (SD), %	12.17 (10.80)	8.64 (7.22)	23.64 (15.13)	15.76 (10.33)	11.86 (10.59)
*Z*-score census tract adult offenses, mean (SD)	0.00 (1.00)	−0.18 (0.71)	−0.11 (0.72)	0.40 (1.39)	0.09 (1.10)
Census tract racial/ethnic diversity, mean (SD)	0.38 (0.18)	0.34 (0.17)	0.43 (0.19)	0.44 (0.18)	0.45 (0.17)

### Procedure

For the ABCD Study, adolescents and their caregivers were interviewed from 2016 (ie, wave 1) to 2023 (ie, wave 5).^48^ The present study used data from waves 2 (2017-2019), 3 (2019-2021), and 4 (2020-2022), when key constructs were available, and because data collection for wave 5 is currently ongoing. The University of Minnesota’s Institutional Review Board exempted the present study from review because the analysis is of deidentified, publicly available data.

### Measures

#### Early adolescents’ pubertal development

At each wave, youth and caregivers completed the Pubertal Development Scale,^49^^,^^50^ which consists of 5 questions regarding changes in height, body hair, skin, voice, and facial hair for male youth or changes in height, body hair, skin, breast development, and menarche for female youth. For each question, youth and caregivers responded with answers on a 4-point Likert scale (1 = has not begun yet, 4 = seems complete), and their responses were then averaged due to high correlations at each wave (*r* range = 0.61-0.77). Notably, distinguishing between youth and parent reports yielded similar results as our main analysis. In line with prior studies,^51^^,^^52^ these means were regressed on age separately by sex to obtain residual scores with higher values indicating more advanced pubertal development relative to same-sex and -age peers.

#### Negative family criminal legal system exposure

At each wave, adolescents reported whether their family members had any contact with the criminal legal system as part of the Life Events Scale.^53^^,^^54^ Adolescents completed 3 items that assessed their families’ contact with the legal system (ie, *“*One of the parents/caregivers went to jail? Someone in the family was arrested? Parents/caregivers got into trouble with the law?*”*; 0 = no, 1 = yes) and rated such contact as a positive or negative event. Following the lead of prior research,^53^ we summed the items that youth rated as negative, so high values on this sum score indicated more negative types of family criminal legal system exposure.

#### Negative family strain

At each wave, adolescents used the Life Events Scale to report strain-related events in the family. Youth reported 5 possible events that happened in their families (ie, *“*Negative change in parents’ financial situation? Mother/father figure lost job? Parents argued more than recently? One parent was away from home more often? Family member had mental/emotional problem?*”*; 0 = no, 1 = yes) and whether such events were positive or negative. The adolescents’ ratings of negative events were sum-scored, with high values indicating more types of negative family strain.

Because family strain is interrelated with family criminal legal system exposure, we investigated whether family criminal legal system exposure was meaningfully distinguishable from family strain. A confirmatory factor analysis involving items for family criminal legal system exposure and family strain indicated that a 2-factor structure fit the data well (χ^2^(13) = 28.38; *P* < .01; root mean square error of approximation [RMSEA] = 0.01; comparative fit index [CFI] = 0.99; standardized root mean square residual [SRMR] = 0.02) and better than a 1-factor structure (Δχ^2^(7) = 446.75, *P* < .001), suggesting that family criminal legal system exposure is distinguishable from and yet related to family strain (see results in Table S1).

#### Covariates

Covariates were assessed at baseline and included youth’s race/ethnicity, age, body mass index, parental education, parental income, 2-parent household status, substance use, other family-related ACEs, direct crime exposure, census-tract percentage of families living below poverty line, census-tract count of total adult offenses (*z* scored to impose a normal distribution for count data), and census-tract racial/ethnic diversity (see Appendix S2 for details).^55^ To specify our results were due to negative (vs positive) criminal legal system exposure, within- and between-person covariates included youths’ ratings of family criminal legal system exposure as positive. With data nested by household for 2996 youth and site (*n* = 21) for all youth, we accounted for household as random effects and site as fixed effects. After we re-estimated our models among 7846 youth without siblings and accounted for site as random effects, our results stayed the same (see Table S2).

### Missing data

Of the 9518 youth who participated in wave 2, 96% participated in wave 3, and 89% participated in wave 4. As detailed in Appendix S3, our data were characterized as conditionally missing at random, warranting our use of full information maximum likelihood to retain all 9518 diverse participants in the present study.^56^

### Analytic plan

All analyses were conducted in *Mplus*, version 8.9,^57^ with maximum likelihood with robust SEs to account for the non-normal distribution of the data. To answer our research questions, we used structural equation multilevel models within a case-crossover design.^58^^,^^59^ These multilevel models were estimated within a hierarchical linear framework assigning time to level 1 and youth to level 2, while accounting for family random effects.^60^ The intraclass correlation coefficients for a 15% randomly selected sub-sample of youth in Table S3 justified our approach because most of the variance for each construct was at the within-youth, between-youth, and between-family levels relative to the between-site level. For this 15% random sub-sample, significant heterogeneity emerged for pubertal development over time, because some youth had increases whereas others had decreases in pubertal development (see Figure S1), enabling us to predict when youth accelerate (vs decelerate) in pubertal development over time. The case-crossover design we used for our multilevel models compares each adolescent with themselves before and after an exposure, which, in the context of our study, refers to negative family criminal legal system exposure. This within-person comparison before and after an adolescent is exposed to an event treats each adolescent as their own control and lessens potential effects of time-invariant confounders that could differ between adolescents on key-study constructs.^58^^,^^59^ To ease interpretation, we person-mean-centered time-varying variables at level 1 and sample-mean-centered time-invariant variables at level 2.^60^

To answer our first research question, we examined whether within-person changes in family criminal legal system exposure predicted within-person changes in the youths’ pubertal development when family strain was absent in the equation. To answer our second research question, we tested whether within-person changes in family criminal legal system exposure predicted within-person changes in youths’ pubertal development via within-person changes in family strain, based on a nonparametric bootstrapping technique to generate SEs for the indirect effect via the multivariate delta method.^61^ For our third research question, we shifted adolescents’ race/ethnicity as a covariate to a grouping variable for a multigroup analysis, in which we tested whether estimates for the multiple racial/ethnic groups could be constrained to be equivalent among each other without causing a significant decrement in model fit. In all models, the false discovery rate method was used to account for multiple hypotheses testing.

## Results

### Descriptive statistics

Table 2 presents mean and SD values for negative family criminal legal system exposure, negative family strain, and youth pubertal development. Across the 3-year period, 6% of youth reported a parent/caregiver got into trouble with the law, 8% reported 1 parent/caregiver went to jail, and 14% reported someone in the family was arrested. One in 6 adolescents (16%) reported 1 or more of these instances. Forty percent of Black youth, 20% of Latinx youth, 16% of Other youth, and 9% of White youth experienced 1 or more negative family criminal legal system exposures. These racial/ethnic differences in family criminal legal system exposure (but not census tract adult-perpetuated offenses) mapped to racial/ethnic differences in pubertal development.

**Table 2:** Means (SDs) of key constructs among full analytic sample and sample by racial/ethnic group.

**Key study variable**	**Full sample (*N* = 9518)**	**White youth (*n* = 5413)**	**Black youth (*n* = 1160)**	**Latinx youth (*n* = 1734)**	**Other youth (*n* = 1211)**
Pubertal development, wave					
2	−0.01 (1.00)	−0.18 (0.93)	0.53 (1.05)	0.13 (0.98)	−0.02 (1.05)
3	−0.02 (1.00)	−0.17 (0.98)	0.39 (0.95)	0.15 (0.98)	0.00 (1.03)
4	−0.02 (1.00)	−0.14 (1.02)	0.25 (0.91)	0.14 (0.94)	0.06 (1.00)
Negative family CLSE, wave					
2	0.14 (0.50)	0.07 (0.36)	0.40 (0.75)	0.16 (0.52)	0.17 (0.57)
3	0.13 (0.48)	0.08 (0.38)	0.32 (0.69)	0.16 (0.54)	0.13 (0.51)
4	0.10 (0.11)	0.06 (0.36)	0.24 (0.61)	0.14 (0.49)	0.12 (0.47)
Negative family strain, wave					
2	0.33 (0.67)	0.29 (0.64)	0.39 (0.70)	0.36 (0.71)	0.35 (0.68)
3	0.33 (0.69)	0.32 (0.68)	0.34 (0.73)	0.36 (0.71)	0.32 (0.66)
4	0.34 (0.70)	0.35 (0.71)	0.25 (0.60)	0.34 (0.70)	0.36 (0.74)

Abbreviation: CLSE, criminal legal system exposure.

### Family criminal legal system exposure and early adolescents’ pubertal development

Table 3 presents multilevel models examining the longitudinal interrelations between negative family criminal legal system exposure and adolescents’ pubertal development within a case-crossover design, after accounting for our covariates and family random effects. An increase in negative family criminal legal system exposure was associated with more advanced pubertal development among early adolescents.

**Table 3:** Unstandardized regression estimates from case-crossover design with structural equation multilevel models predicting early adolescents’ pubertal development across a 3-year period, controlling for covariates, sites as fixed effects,^a^ and family random effects.

	**Baseline model**		**Mediation model**			
	**Pubertal development**		**Negative family strain**		**Pubertal development**	
**Predictor**	**β (SE)**	**95% CI**	**β (SE)**	**95% CI**	**β (SE)**	**95% CI**
Within-person fixed effects						
Time	0.01 (0.00)^**^	0.01-0.02	0.01 (0.00)^**^	0.01-0.02	0.01 (0.00)^*^	0.00-0.02
Negative family CLSE	0.03 (0.01)^*^	0.01-0.05	0.26 (0.02)^***^	0.22-0.30	0.01 (0.01)	−0.01 to 0.04
Negative family strain					0.04 (0.01)^***^	0.03-0.06
Positive family CLSE	0.00 (0.03)	−0.04 to 0.03	0.09 (0.04)	0.00-0.17	0.00 (0.03)	−0.04 to 0.03
Between-person fixed effects						
Black (vs White) youth	0.23 (0.03)^***^	0.18-0.29	−0.13 (0.02)^***^	−0.17 to −.09	0.23 (0.03)^***^	0.18-0.29
Latinx (vs White) youth	0.12 (0.02)^***^	0.07-0.16	−0.04 (0.02)^*^	−0.08 to 0.01	0.12 (0.02)^***^	0.07-0.16
Other (vs White) youth	0.08 (0.02)^***^	0.03-0.13	−0.02 (0.02)	−0.06 to 0.01	0.08 (0.02)^***^	0.03-0.13
Male (vs female) youth	−0.88 (0.01)^***^	−0.91 to −0.85	−0.04 (0.01)^***^	−0.06 to −0.02	−0.88 (0.01)^***^	−0.91 to −0.85
Youth age	−0.01 (0.01)	−0.03 to 0.02	0.01 (0.01)	−0.01 to 0.03	−0.01 (0.01)	−0.03-0.02
Youth BMI	0.04 (0.00)^***^	0.04-0.05	0.01 (0.00)	0.00-0.01	0.04 (0.00)^***^	0.04-0.05
Parental education	−0.01 (0.00)	−0.02 to −0.01	0.00 (0.00)	−0.01 to 0.01	−0.01 (0.00)	−0.02 to −0.01
Parental income	−0.01 (0.01)	−0.02 to 0.01	−0.01 (0.00)^**^	−0.02 to −0.01	−0.01 (0.01)	−0.02 to 0.01
Two (vs single) parent household	−0.07 (0.02)^**^	−0.11 to −0.03	−0.07 (0.02)^***^	−0.11 to 0.04	−0.07 (0.02)^**^	−0.11 to –0.03
Youth’s substance use	0.06 (0.02)^***^	0.03-0.09	0.05 (0.01)^***^	0.03-0.08	0.06 (0.02)^***^	0.03-0.09
Family-related adversities	0.05 (0.01)^***^	0.04-0.07	0.09 (0.01)^***^	0.08-0.10	0.05 (0.01)^***^	0.04-0.07
Direct crime exposure	0.03 (0.02)	−0.01 to 0.06	0.08 (0.01)^***^	0.06-0.11	0.03 (0.02)	−0.01 to 0.06
Census tract live below poverty, %	0.00 (0.00)	−0.01 to 0.01	−0.01 (0.00)^***^	−0.01 to −0.01	0.00 (0.00)	−0.01 to 0.01
*Z* score census tract adult offenses	−0.01 (0.02)	−0.06 to 0.04	−0.02 (0.02)	−0.05 to 0.02	−0.01 (0.02)	−0.06 to 0.04
Census tract racial/ethnic diversity	0.14 (0.05)^**^	0.05-0.23	0.05 (0.03)	−0.01 to 0.11	0.14 (0.05)^**^	0.05-0.23
Negative family CLSE	0.01 (0.02)	−0.03 to 0.05	0.34 (0.02)^***^	0.30-0.38	0.01 (0.02)	−0.03 to 0.05
Positive family CLSE	0.14 (0.08)	−0.01 to 0.28	0.15 (0.07)	0.00-0.29	0.14 (0.08)	−0.01 to 0.28
Intercepts	−0.30 (0.17)	−0.64 to 0.04	0.23 (0.12)	−0.01 to 0.48	−0.30 (0.17)	−0.64 to 0.04
Within-person residual variances	0.24 (0.00)^***^	0.23-0.24	0.30 (0.01)^***^	0.29-0.31	0.24 (0.00)^***^	0.23-0.24
Between-person residual variances	0.34 (0.01)^***^	0.32-0.35	0.13 (0.01)^***^	0.11-0.14	0.34 (0.01)^***^	0.32-0.35

Abbreviations: BMI, body mass index; CLSE, criminal legal system exposure.

^*^ *P* < .05, ^**^P < .01, ^***^P < .001, after we used the Benjamini-Hochberg method to account for multiple testing.

a Fixed effects of sites on adolescents’ pubertal development and negative family strain are reported in Table S5.

### The mediating role of negative family strain

Table 3 also presents results from multilevel models examining the longitudinal interrelations among negative family legal system exposure, family strain, and early adolescents’ pubertal development within a case-crossover design, after accounting for our covariates and family random effects. In waves when youth were exposed to more negative family criminal legal system exposure, youth reported more negative family strain. In turn, negative family strain was associated with increases in adolescents’ advanced pubertal development. The indirect effect of family criminal legal system exposure on adolescents’ pubertal development via family strain was significant (β = .01; SE = 0.00; 95% CI, 0.01-0.02; *P* < .001). After accounting for family strain, family criminal legal system exposure was nonsignificantly related to adolescent pubertal development, positioning family strain as a full mediator. The effect sizes for family criminal legal system exposure on family strain and pubertal development were small (β = .15 and .02, respectively), which prior scholars have argued are meaningful.^62^ The final model fit the data well (χ^2^(1) = 1.89; *P* < .01; RMSEA = 0.01; CFI = 1.00; Tucker-Lewis Index = 0.98; SRMR_within_ = 0.01 and SRMR_between_ = 0.01).

### The role of race/ethnicity

After we used race/ethnicity as a grouping variable for multigroup analyses, constraining the estimates to be equivalent across groups did not result in a significant decrement in model fit (Δχ^2^(6) = 9.46; *P* = .15), suggesting that race/ethnicity did not significantly moderate our results.

### Sensitivity analyses

We ran several sensitivity analyses to assess the robustness of our inferences. First, mediation requires that a mediator temporally follows the independent variable and precedes the dependent variable^61^; therefore, we tested whether negative family criminal legal system exposure predicted family strain 1 wave later and whether family strain predicted pubertal development 1 wave thereafter, controlling for prior family strain and pubertal development in path analyses. The data in Table 4 indicate negative family criminal legal system exposure was associated with more family strain 1 wave later, and family strain was associated with more advanced adolescent pubertal development 1 wave thereafter. The indirect effect of negative family criminal legal system exposure on pubertal development via family strain was significant (β = .01; SE = 0.00; 95% CI, 0.01-0.01; *P* < .05), and the direct effect of negative family criminal legal system exposure on pubertal development was nonsignificant, supporting the finding that family strain was a full mediator.

**Table 4:** Unstandardized regression estimates from path analyses predicting wave-3 family strain and wave-4 early adolescents’ pubertal development, controlling for covariates, sites as fixed effects,^a^ and family random effects.

**Predictor**	**Wave 3 negative family strain**		**Wave 4 pubertal development**	
	**β (SE)**	**95% CI**	**β (SE)**	**95% CI**
Wave 2 negative family CLSE	0.08 (0.03)^**^	0.01-0.14	0.00 (0.02)	−0.02 to 0.02
Wave 3 negative family strain			0.03 (0.01)^**^	0.01-0.05
Wave 2 outcome	0.43 (0.02)^***^	0.38-0.47	0.47 (0.01)^***^	0.45-0.49
Black (vs White) youth	−0.15 (0.06)	−0.28 to −0.03	0.01 (0.03)	−0.05 to 0.08
Latinx (vs White) youth	−0.07 (0.05)	−0.18 to 0.03	0.12 (0.03)^***^	0.06-0.17
Other (vs White) youth	−0.08 (0.05)	−0.17 to 0.02	0.08 (0.03)^**^	0.03-0.13
Male (vs female) youth	−0.11 (0.03)^***^	−0.17 to −0.05	−0.71 (0.02)^***^	−0.74 to −0.67
Youth age	0.08 (0.03)^**^	0.02-0.14	0.01 (0.02)	−0.02 to 0.05
Youth BMI	0.00 (0.00)	−0.01 to 0.01	0.02 (0.00)^***^	0.01-0.02
Parental education	0.00 (0.01)	−0.02 to 0.02	0.00 (0.01)	−0.01 to 0.01
Parental income	−0.01 (0.01)	−0.03 to 0.01	0.00 (0.01)	−0.01 to 0.02
Two (vs single) parent household	−0.19 (0.04)^***^	−0.27 to −0.11	−0.03 (0.02)	−0.07 to 0.02
Youth’s substance use	0.07 (0.03)	0.00-0.14	0.05 (0.02)	0.01-0.08
Family-related adversities	0.05 (0.02)	0.01-0.09	0.00 (0.01)	−0.02 to 0.03
Direct crime exposure	−0.01 (0.03)	−0.08 to 0.06	0.03 (0.02)	0.00-0.07
Census tract live below poverty, %	−0.01 (0.00)^***^	−0.01 to −0.01	0.00 (0.00)	0.00-0.00
Z score census tract adult offenses	−0.05 (0.06)	−0.16 to 0.07	0.01 (0.03)	−0.05 to 0.07
Census tract racial/ethnic diversity	0.08 (0.10)	−0.12 to 0.28	0.04 (0.05)	−0.06 to 0.14
Wave 2 positive family CLSE	0.01 (0.05)	−0.08 to 0.11	−0.04 (0.02)	−0.09 to −0.01
Intercepts	0.18 (0.16)	−0.14 to 0.50	−0.27 (0.19)	−0.64 to 0.10
Random effects	0.41 (0.01)^***^	0.39-0.44	0.43 (0.01)^***^	0.41-0.44

Abbreviation: BMI, body mass index; CLSE, criminal legal system exposure.

^**^ *P* < .01, ^***^P < .001, after we used the Benjamini-Hochberg method to account for multiple testing.

a Fixed effects of sites on adolescents’ pubertal development and negative family strain are reported in Table S6.

Second, given that male and female youth responded to distinct measures of pubertal development, we tested whether our results differed by sex, using multigroup analyses with sex as the grouping variable. We found that we could constrain key paths to be equivalent across sexes without causing a significant in decrement in model fit (Δχ^2^(2) = 1.14; *P* = .56), suggesting that our results were the same between male and female youth.

Third, after we adjusted for confounders that do not change over time and that are linked to racism, such as family and neighborhood socioeconomic status, our findings did not differ across racial/ethnic groups and raised questions about whether socioeconomic status interacted with race/ethnicity to modify our results. Based on descriptive zero-order bivariate correlations, Table S4 shows associations between socioeconomic status and our key constructs (ie, family criminal legal system exposure, family strain, and pubertal development) at each wave separately for each racial/ethnic group. Socioeconomic status was protective against advanced pubertal development and negative family strain among White youth, less frequently protective against these outcomes among Latinx and Other youth, and unrelated to these outcomes among Black youth, illustrating that the benefits associated with socioeconomic status are not equally shared across racial/ethnic groups.

To test whether socioeconomic status interacted with race/ethnicity in our structural equation multilevel model, we regressed family strain and pubertal development on interaction terms, taking the product of sample-mean-centered terms for family criminal legal system exposure and each socioeconomic status indicator (ie, parental education, income, marital status, and census-tract percentage of families living below the poverty line) at the between-person level. Thereafter, we tested whether these coefficients could be constrained to be equal across racial/ethnic groups without causing a significant decrement in model fit. Ultimately, we could add such constraints (Δχ^2^(12) = 15.85; *P* = .20) suggesting that race/ethnicity and socioeconomic status did not interact with each other to modify associations between our key constructs.

## Discussion

Family criminal legal system exposure is prevalent: we found that 1 in 6 adolescents experienced 1 or more negative family criminal legal system exposures during the present 3-year study. This rate is likely a conservative estimate, because participants in the ABCD Study generally reflect more socially privileged youth than the general US population. Youth of color experienced more negative family criminal legal system exposure, despite Black and White youth not differing from each other on their exposures to adult-perpetuated offenses in their census tracts. This counterintuitive result is reminiscent of institutional practices that disadvantage particular social groups; our descriptive findings showed social characteristics, such as race/ethnicity (but not crime), predicted greater legal system exposure.^63^

In our longitudinal analyses, negative family criminal legal system exposure predicted greater family strain, which, in turn, predicted more advanced pubertal development among adolescents. These findings fill a gap in the existing literature and help identify potential contributors of adolescents’ pubertal development. The present findings potentially stem from how parental imprisonment can tarnish parents’ employment status and have proliferating consequences (eg, health insurance loss, limited future job prospects). In addition, family members’ imprisonment and arrests can limit youth’s perceived opportunities to succeed.^14^ Following criminal legal system exposure, families receive little to no resources after parental imprisonment and during police stop-and-search procedures in adolescents’ presence.^64^ These adversities during early adolescence may have contributed to youths’ stress internalization and accelerated pubertal development. In addition to corroborating findings from prior studies that focused on youth born into single-parent households^6^ and those with incarcerated parents,^25^^,^^65^ the present study enhances extant findings’ generalizability with a relatively more representative sample of youth.

Although the effects of family criminal legal system exposure did not differ across racial/ethnic groups, the rate of family criminal legal system exposure was concentrated among families of color. The detrimental effects of negative family criminal legal system exposure across racial/ethnic groups illustrate the universal harms that occur when youth negatively witness or learn about their family members’ imprisonment, arrest, or trouble with the law, which is in line with findings from prior studies.^6^^,^^8^^,^^66^^,^^67^ In response to these negative encounters, youth, regardless of background, will share some degree of psychosocial stress that may accelerate their biological age. However, the nonsignificant racial/ethnic group differences in the impacts of family criminal legal system exposure should not detract from the alarming racial/ethnic group differences in rates of such exposures. Youth of color had twice as many negative family criminal legal system exposures as White youth, suggesting that the negative effects of family criminal legal system exposure are disproportionately experienced across racial/ethnic groups. Moreover, as we found in our sensitivity analysis, socioeconomic status had diminished returns among youth of color, primarily Black youth. Altogether, above and beyond differences associated with socioeconomic status, more youth of color are exposed to health risks than White youth, and the criminal legal system is 1 source of these health inequities.

The present study’s limitations provide directions for future research. First, the cohort was representative of more socioeconomically affluent families relative to the national average, which may suggest that our findings may be weaker relative to extant studies; families that are more socioeconomically affluent may have more resources to cope with stress from criminal legal system exposure.^9^ Conversely, the present findings may be stronger relative to extant studies, because the novel aspect of stress among socioeconomically affluent families may not have enabled them to learn about which practices provide them with resilience to cope with such stress.^21^ Second, the present measure of family criminal legal system exposure may have compounded family members’ imprisonment with their criminal behaviors.^68^ Nonetheless, we included time-invariant covariates (eg, direct crime exposure, neighborhood-level crime) to reduce the effects of such confounders. Third, we did not measure criminal legal system exposure of vicarious peers,^69^ neighbors,^70^ or unfamiliar adults.^71^ Scholars should explore these possibilities in future research.

## Conclusion

In the present study, we used a national and longitudinal sample of US families to understand rates of family criminal legal system exposures and their connections to adolescent pubertal development, which is a public health crisis.^41^^,^^42^ Youth are undergoing puberty at younger ages today than in the past, and such precocious puberty has detrimental consequences on the life courses of adolescents,^72^^,^^73^ positioning family criminal legal system exposures as likely social determinants of health that disproportionately affect youth of color.^6^ It is vital that scholars guide pediatricians, partner with public health practitioners, and inform policymakers about the unintended consequences of criminal legal system exposure in adolescents’ lives.

## Supplementary Material

Web_Material_kwae457

## Data Availability

Data used in the preparation of this article were obtained from the ABCD Study (https://abcdstudy.org), held in the National Institute of Mental Health Data Archive.
