# Supplementary material for: Family criminal legal system exposure and early adolescents’ pubertal development: the mediating role of family strain
Source: Am J Epidemiol. 2024 Dec 13;194(10):2870–8. doi: 10.1093/aje/kwae457 (PMC12527266; doi:10.1093/aje/kwae457)
Supplement: Web_Material_kwae457 [file web_material_kwae457.docx]

**Supplementary Material**

**Family Criminal Legal System Exposure and Early Adolescents’ Pubertal Development: The Mediating Role of Family Strain**

Juan Del Toro, Michael Roettger, Dylan B. Jackson, & Sylia Wilson

**Contents:**

| **Appendix S1** | Exclusionary criteria |
| --- | --- |
| **Appendix S2** | Covariates |
| **Appendix S3** | Missing data |
| **Table S1** | A confirmatory factor analysis with items from the life events scale |
| **Table S2** | Structural equation multilevel models predicting early adolescents’ pubertal development across a three-year period, excluding siblings and controlling for sites as random effects |
| **Table S3** | Intraclass correlation coefficients (iccs) from a 15%-random subset of the present study |
| **Table S4** | Zero-order bivariate correlations between socioeconomic-status indicators and key constructs by racial/ethnic group |
| **Table S5** | Structural equation multilevel models predicting early adolescents’ pubertal development across a three-year period, controlling for covariates, sites as fixed effects, and family random effects |
| **Table S6** | Unstandardized regression estimates from path analyses predicting wave-3 family strain and wave-4 early adolescents’ pubertal development, controlling for covariates, sites as fixed effects, and family random effects |
| **Figure S1** | A visual of each youth’s trajectory of their pubertal development score across three waves |

**Appendix S1: Exclusionary Criteria**

Of the initial 11,868 youth who participated in ABCD at baseline, 356 youth did not complete any of the questions on family criminal legal system exposure across the three waves and 10 youth did not self-report nor have parent-reported youth’s pubertal-physical maturation. Of the remaining 11,502 early adolescents, 1,984 had missing data across the covariates listed in our main manuscript. Between the 9,518 of included early adolescents and their 1,984 excluded peers, included-early adolescents were more likely to be White (than Black and Latinx; *χ^2^*(3) = 3.43, *p* = .33), come from households with higher parental education status and parental income [*t*(2526) = 20.13, *p* < .001; *t*(1175) = 13.14, *p* < .001, respectively], and live in neighborhoods with lower adult-perpetrated violence [*t*(1624) = 7.02, *p* < .001] and less concentrations of families living below poverty [*t*(1619) = 16.57, *p* < .001]. No relations emerged between included versus excluded early adolescents and their sex (*χ^2^*(1) = 0.05, *p* = .83) and age [*t*(1150) = 1.65, *p* = .10]. After accounting for these demographic variables and mean-aggregating our key constructs (i.e., family criminal legal system exposure, family strain, and pubertal development) across the three waves, semi-partial correlations indicated no associations between study participation and family criminal legal system exposure (*r* = .00, *p* < .79), family strain (*r* = .01, *p* = .32), and adolescents’ pubertal development (*r* = .00, *p* = .99).

**Appendix S2: Covariates**

We accounted for several potential third variable confounds that could bias the link between negative family criminal legal system exposure and youth’s pubertal development. All covariates were assessed at baseline and included youth’s race, age (range=9-11), body mass index (BMI), parental education (0=*never attended/kindergarten only*, 21=*doctoral degree*), parental income (1=*less than $5,000*; 10=*$200,000 and greater*), two-parent household status (0=*widowed, divorced, separated, or never married*, 1=*married or living partner*),substance use (two-item; e.g., “You have ever tried a puff from tobacco?”), other family-related adverse childhood experiences (four items; e.g., “Family member had drug and/or alcohol related problem?”), direct exposure to crime (four-item; e.g., “Was a victim to crime/violence/assault?”), z-scored residential history (i.e., census tract) of adults’ total infarctions, and residential history of percentage of families below poverty line. In addition, considering the data were nested by household for 2,996 youth and site (*N*=21) for all youth, we accounted for nesting by household using TYPE=COMPLEX and site as fixed effects. In supplemental analyses, we re-estimated our models focusing on only 7,846 with a single participant per household and accounted for site as random effects using TYPE=COMPLEX; ultimately, our results were the same (see Table S5).

**Appendix S3: Missing Data**

Of the 9,518 in Wave-2, 96% participated in Wave-3 and 89% participated in Wave-4. Across the three waves, 88% of the sample completed all three assessments, 9% completed two assessments, and 3% completed only one assessment. After adjusting for multiple testing use the FDR method, White and Other youth on average completed the most waves followed by Latinx youth, and Black youth on average completed the least waves [*F*(4, 9514)=29.46, *p*<.001]. In addition, youth’s participation did not differ by sex [*t*(9273)=1.61, *p*=.11) nor chronological age (*r*=0.01, *p*=.31), but youth who completed more waves came from households with higher educational attainment and income among caregivers (*r*=.12, *p*<.001; *r*=.11, *p*<.001) and lived in less socioeconomically disadvantaged neighborhoods (adult-perpetrated offenses: *r*=-.03, *p*<.01; neighbors live below poverty: *r*=-.09, *p*<.001). After we adjusted for these demographic variables, partial correlations indicated that youth’s participation was unrelated to pubertal development at our initial assessment of key constructs, including early adolescents’ pubertal development (*r*=-.01, *p*=.18), negative family criminal legal system exposure (*r*=-.02, *p*=.07), and negative family strain (*r*=-.01, *p*=.27).

| **Table S1**  *A Confirmatory Factor Analysis with Items from the Life Events Scale* | | | | | |
| --- | --- | --- | --- | --- | --- |
| Factor loadings | | |  | | *B (SE)* |
| Factor 1 | | |  | |  |
|  | One of the parents/caregivers went to jail | | | | 1.00 (0.00)*** |
|  | Parent/caregiver got into trouble with the law | | | | 0.84 (0.02)*** |
|  | Someone in the family was arrested | | | | 0.89 (0.02)*** |
| Factor 2 | | |  | |  |
|  | Negative change in parents’ financial situation | | | | 1.00 (0.00)*** |
|  | Mother/father figure lost job | | | | 0.91 (0.05)*** |
|  | Parents argued more than previously | | | | 0.99 (0.06)*** |
|  | One parent was away from home often | | | | 0.79 (0.05)*** |
|  | Family member had mental/emotional problem | | | | 0.76 (0.05)*** |
| Covariances | | |  | |  |
|  | | Factor 1 WITH Factor 2 | | | 0.36 (0.02)*** |
| Thresholds | | |  | |  |
|  | | One of the parents/caregivers went to jail | | | 1.47 (0.02)*** |
|  | | Parent/caregiver got into trouble with the law | | | 1.66 (0.02)*** |
|  | | Someone in the family was arrested | | | 1.19 (0.02)*** |
|  | | Negative change in parents’ financial situation | | | 1.42 (0.02)*** |
|  | | Mother/father figure lost job | | | 1.30 (0.02)*** |
|  | | Parents argued more than previously | | | 1.35 (0.02)*** |
|  | | One parent was away from home often | | | 0.74 (0.01)*** |
|  | | Family member had mental/emotional problem | | | 1.31 (0.02)*** |
| Variances | | | |  |  |
|  | Factor 1 | | | | 0.95 (0.02)*** |
|  | Factor 2 | | | | 0.39 (0.03)*** |

| **Table S2**  *Structural Equation Multilevel Models Predicting Early Adolescents’ Pubertal Development Across a Three-Year Period, Excluding Siblings and Controlling for Sites as Random Effects* | | | | |
| --- | --- | --- | --- | --- |
|  | |  | Family strain | Pubertal development |
| Predictors | |  | *B (SE)* | *B (SE)* |
| Within-person fixed effects | |  |  |  |
|  | Time | | 0.01 (0.01) | 0.01 (0.01) |
|  | Negative family criminal legal system exposure | | 0.25 (0.01)*** | 0.02 (0.01) |
|  | Negative family strain | | -- | 0.04 (0.01)*** |
|  | Positive family criminal legal system exposure | | 0.09 (0.05) | 0.00 (0.04) |
| Between-person fixed effects | |  |  |  |
|  | Black (vs. White) youth | | -0.14 (0.02)*** | 0.28 (0.04)*** |
|  | Latinx (vs. White) youth | | -0.03 (0.02) | 0.14 (0.04)** |
|  | Other (vs. White) youth | | -0.03 (0.02) | 0.10 (0.02)*** |
|  | Male (vs. female) youth | | -0.05 (0.01)*** | -0.90 (0.02)*** |
|  | Youth age | | 0.01 (0.01) | -0.03 (0.01) |
|  | Youth BMI | | 0.00 (0.00) | -0.03 (0.02)*** |
|  | Parental education | | 0.00 (0.00) | 0.04 (0.00)*** |
|  | Parental income | | -0.01 (0.00)** | 0.00 (0.01) |
|  | Two (vs. single) parent household | | -0.08 (0.02)*** | -0.01 (0.01) |
|  | Youth’s substance use | | 0.05 (0.01)** | -0.07 (0.02)** |
|  | Family-related adversities | | 0.09 (0.01)*** | 0.07 (0.02)*** |
|  | Direct crime exposure | | 0.08 (0.01)*** | 0.04 (0.01)** |
|  | Census tract live below poverty | | -0.01 (0.00)*** | 0.00 (0.00) |
|  | Z-score census tract adult offenses | | 0.01 (0.01) | -0.02 (0.02) |
|  | Census tract racial/ethnic diversity | | 0.10 (0.04)* | 0.14 (0.06)* |
|  | Negative family criminal legal system exposure | | 0.33 (0.03)*** | 0.01 (0.02) |
|  | Positive family criminal legal system exposure | | 0.11 (0.07) | 0.11 (0.09) |
| *Note*. * *p* < .05, ** *p* < .01, *** *p* < .001, after we used the Benjamini-Hochberg method to account for multiple testing. | | | | |

| **Table S3**  *Intraclass Correlation Coefficients (ICCs) From a 15%-Random Subset of the Present Study* | | | | |
| --- | --- | --- | --- | --- |
| Key study variables | Within-person | Between-person | Between-family | Between-site |
| Pubertal development categories (youth report) | .49 | .15 | .34 | .02 |
| Pubertal development categories (parent report) | .45 | .24 | .28 | .02 |
| Negative family criminal legal system exposure | .49 | .09 | .41 | .01 |
| Negative family strain | .62 | .06 | .31 | .01 |

| **Table S4**  *Zero-Order Bivariate Correlations between Socioeconomic-Status Indicators and Key Constructs by Racial/Ethnic Group* | | | | | | | | | |
| --- | --- | --- | --- | --- | --- | --- | --- | --- | --- |
| Socioeconomic status indicators | Pubertal development Wave 2 | Pubertal development Wave 3 | Pubertal development Wave 4 | Negative family CLSE Wave 2 | Negative family CLSE Wave 3 | Negative family CLSE Wave 4 | Negative family strain Wave 2 | Negative family strain Wave 3 | Negative family strain Wave 4 |
| White youth (*n* = 5,413) | | | | | | | | | |
| Parental education | -.11** | -.07** | -.06** | -.18** | -.19** | -.15** | -.12** | -.09** | -.07** |
| Parental income | -.10** | -.06** | -.04** | -.21** | -.22** | -.18** | -.14** | -.14** | -.12** |
| Two (vs. single) parent house | -.08** | -.06** | -.05** | -.15** | -.16** | -.14** | -.14** | -.17** | -.11** |
| %Census tract below poverty | .06** | .06** | .04** | .08** | .08** | .05** | .06** | .03* | .03* |
| Black youth (*n* = 1,160) | | | | | | | | | |
| Parental education | -.02 | -.01 | .04 | -.17** | -.14** | -.10** | -.01 | -.01 | .02 |
| Parental income | -.01 | .02 | .05 | -.17** | -.14** | -.14** | -.05 | -.05 | -.03 |
| Two (vs. single) parent house | -.02 | -.03 | -.02 | -.10** | -.09** | -.09** | -.04 | -.04 | .02 |
| %Census tract below poverty | -.01 | -.03 | -.06* | .12** | .06* | .10** | .02 | .00 | .00 |
| Latinx youth (*n* = 1,734) | | | | | | | | | |
| Parental education | -.03 | -.04* | .00 | -.11** | -.08** | -.06** | .00 | -.01 | .03 |
| Parental income | -.08** | -.07** | -.02 | -.13** | -.11** | -.09** | -.05* | -.05* | -.01 |
| Two (vs. single) parent house | -.05* | -.02 | -.03 | -.11** | -.10** | -.11** | -.07** | -.08** | -.04 |
| %Census tract below poverty | .05* | .04 | .02 | .10** | .08** | .04 | -.01 | -.02 | -.06** |
| Other youth (*n* = 1,211) | | | | | | | | | |
| Parental education | -.22** | -.14** | -.09** | -.23** | -.20** | -.16** | -.14** | -.06* | -.01 |
| Parental income | -.24** | -.16** | -08** | -.28** | -.23** | -.16** | -.18** | -.08** | -.05 |
| Two (vs. single) parent house | -.15** | -.12** | -.07* | -.21** | -.16** | -.11** | -.14** | -.06* | -.01 |
| %Census tract below poverty | .21** | .15** | .09** | .17** | .14** | .07* | .08** | .01 | .00 |
| *Note*. * *p* < .05, ** *p* < .01. *CLSE = Criminal legal system exposure.* | | | | | | | | | |

| **Table S5**  *Structural Equation Multilevel Models Predicting Early Adolescents’ Pubertal Development Across a Three-Year Period, Controlling for Covariates, Sites as Fixed Effects, and Family Random Effects* | | | | | | | |
| --- | --- | --- | --- | --- | --- | --- | --- |
|  | |  | Baseline model |  | Mediation model | |  |
|  | |  | Pubertal development | | Family strain | Pubertal development | |
| Predictors | |  | *B (SE)* | | *B (SE)* | *B (SE)* | |
| Within-person fixed effects | |  |  | |  |  | |
|  | Time | | 0.01 (0.00)** | | 0.01 (0.00)** | 0.01 (0.00)* | |
|  | Negative family CLSE | | 0.03 (0.01)* | | 0.26 (0.02)*** | 0.01 (0.01) | |
|  | Negative family strain | | -- | | -- | 0.04 (0.01)*** | |
|  | Positive family CLSE | | 0.00 (0.03) | | 0.09 (0.04) | 0.00 (0.03) | |
| Between-person fixed effects | |  |  | |  |  | |
|  | Black (vs. White) youth | | 0.23 (0.03)*** | | -0.13 (0.02)*** | 0.23 (0.03)*** | |
|  | Latinx (vs. White) youth | | 0.12 (0.02)*** | | -0.04 (0.02)* | 0.12 (0.02)*** | |
|  | Other (vs. White) youth | | 0.08 (0.02)*** | | -0.02 (0.02) | 0.08 (0.02)*** | |
|  | Male (vs. female) youth | | -0.88 (0.01)*** | | -0.04 (0.01)*** | -0.88 (0.01)*** | |
|  | Youth age | | -0.01 (0.01) | | 0.01 (0.01) | -0.01 (0.01) | |
|  | Youth BMI | | 0.04 (0.00)*** | | 0.01 (0.00) | 0.04 (0.00)*** | |
|  | Parental education | | -0.01 (0.00) | | 0.00 (0.00) | -0.01 (0.00) | |
|  | Parental income | | -0.01 (0.01) | | -0.01 (0.00)** | -0.01 (0.01) | |
|  | Two (vs. single) parent household | | -0.07 (0.02)** | | -0.07 (0.02)*** | -0.07 (0.02)** | |
|  | Youth’s substance use | | 0.06 (0.02)*** | | 0.05 (0.01)*** | 0.06 (0.02)*** | |
|  | Family-related adversities | | 0.05 (0.01)*** | | 0.09 (0.01)*** | 0.05 (0.01)*** | |
|  | Direct crime exposure | | 0.03 (0.02) | | 0.08 (0.01)*** | 0.03 (0.02) | |
|  | %Census tract live below poverty | | 0.00 (0.00) | | -0.01 (0.00)*** | 0.00 (0.00) | |
|  | Z-score census tract adult offenses | | -0.01 (0.02) | | -0.02 (0.02) | -0.01 (0.02) | |
|  | Census tract racial/ethnic diversity | | 0.14 (0.05)** | | 0.05 (0.03) | 0.14 (0.05)** | |
|  | Negative family CLSE | | 0.01 (0.02) | | 0.34 (0.02)*** | 0.01 (0.02) | |
|  | Positive family CLSE | | 0.14 (0.08) | | 0.15 (0.07) | 0.14 (0.08) | |
|  | Site 2 (vs. Site 1) | | 0.06 (0.10) | | -0.01 (0.08) | 0.06 (0.10) | |
|  | Site 3 (vs. Site 1) | | 0.22 (0.11) | | -0.05 (0.08) | 0.22 (0.11) | |
|  | Site 4 (vs. Site 1) | | 0.03 (0.10) | | -0.07 (0.07) | 0.03 (0.10) | |
|  | Site 5 (vs. Site 1) | | 0.15 (0.11) | | -0.07 (0.08) | 0.15 (0.11) | |
|  | Site 6 (vs. Site 1) | | 0.04 (0.10) | | -0.03 (0.08) | 0.04 (0.10) | |
|  | Site 7 (vs. Site 1) | | 0.17 (0.10) | | -0.10 (0.08) | 0.17 (0.10) | |
|  | Site 8 (vs. Site 1) | | 0.00 (0.10) | | -0.01 (0.08) | 0.00 (0.10) | |
|  | Site 9 (vs. Site 1) | | 0.15 (0.05)** | | 0.08 (0.04) | 0.15 (0.05)** | |
|  | Site 10 (vs. Site 1) | | -0.07 (0.08) | | 0.02 (0.06) | -0.07 (0.08) | |
|  | Site 11 (vs. Site 1) | | 0.19 (0.11) | | -0.03 (0.08) | 0.19 (0.11) | |
|  | Site 12 (vs. Site 1) | | 0.14 (0.10) | | -0.08 (0.08) | 0.14 (0.10) | |
|  | Site 13 (vs. Site 1) | | 0.07 (0.10) | | -0.06 (0.07) | 0.07 (0.10) | |
|  | Site 14 (vs. Site 1) | | 0.04 (0.10) | | -0.10 (0.08) | 0.04 (0.10) | |
|  | Site 15 (vs. Site 1) | | 0.10 (0.10) | | -0.09 (0.07) | 0.10 (0.10) | |
|  | Site 16 (vs. Site 1) | | -0.11 (0.10) | | -0.04 (0.07) | -0.11 (0.10) | |
|  | Site 17 (vs. Site 1) | | 0.12 (0.11) | | -0.09 (0.08) | 0.12 (0.11) | |
|  | Site 18 (vs. Site 1) | | 0.06 (0.10) | | -0.07 (0.07) | 0.06 (0.10) | |
|  | Site 19 (vs. Site 1) | | 0.10 (0.11) | | -0.13 (0.08) | 0.10 (0.11) | |
|  | Site 20 (vs. Site 1) | | 0.02 (0.10) | | -0.09 (0.07) | 0.02 (0.10) | |
|  | Site 21 (vs. Site 1) | | 0.09 (0.10) | | -0.09 (0.07) | 0.09 (0.10) | |
| *Note*. * *p* < .05, ** *p* < .01, *** *p* < .001, after we used the Benjamini-Hochberg FDR method to account for multiple testing. *CLSE = Criminal legal system exposure.* | | | | | | | |

| **Table S6**  *Unstandardized Regression Estimates from Path Analyses Predicting Wave-3 Family Strain and Wave-4 Early Adolescents’ Pubertal Development, Controlling for Covariates, Sites as Fixed Effects, and Family Random Effects* | | | | | |
| --- | --- | --- | --- | --- | --- |
|  | |  | Wave-3 negative family strain |  | Wave-4 pubertal development |
| Predictors | |  | *B (SE)* | | *B (SE)* |
|  | Wave-2 negative family CLSE | | 0.08 (0.03)** | | 0.00 (0.02) |
|  | Wave-3 negative family strain | | -- | | 0.03 (0.01)** |
|  | Wave-2 outcome | | 0.43 (0.02)*** | | 0.47 (0.01)*** |
|  | Black (vs. White) youth | | -0.15 (0.06) | | 0.01 (0.03) |
|  | Latinx (vs. White) youth | | -0.07 (0.05) | | 0.12 (0.03)*** |
|  | Other (vs. White) youth | | -0.08 (0.05) | | 0.08 (0.03)** |
|  | Male (vs. female) youth | | -0.11 (0.03)*** | | -0.71 (0.02)*** |
|  | Youth age | | 0.08 (0.03)** | | 0.01 (0.02) |
|  | Youth BMI | | 0.00 (0.00) | | 0.02 (0.00)*** |
|  | Parental education | | 0.00 (0.01) | | 0.00 (0.01) |
|  | Parental income | | -0.01 (0.01) | | 0.00 (0.01) |
|  | Two (vs. single) parent household | | -0.19 (0.04)*** | | -0.03 (0.02) |
|  | Youth’s substance use | | 0.07 (0.03) | | 0.05 (0.02) |
|  | Family-related adversities | | 0.05 (0.02) | | 0.00 (0.01) |
|  | Direct crime exposure | | -0.01 (0.03) | | 0.03 (0.02) |
|  | %Census tract live below poverty | | -0.01 (0.00)*** | | 0.00 (0.00) |
|  | Z-score census tract adult offenses | | -0.05 (0.06) | | 0.01 (0.03) |
|  | Census tract racial/ethnic diversity | | 0.08 (0.10) | | 0.04 (0.05) |
|  | Wave-2 positive family CLSE | | 0.01 (0.05) | | -0.04 (0.02) |
|  | Site 2 (vs. Site 1) | | -0.26 (0.35) | | 0.06 (0.79) |
|  | Site 3 (vs. Site 1) | | -0.16 (0.36) | | 0.07 (0.79) |
|  | Site 4 (vs. Site 1) | | -0.38 (0.35) | | 0.05 (0.79) |
|  | Site 5 (vs. Site 1) | | -0.22 (0.36) | | 0.17 (0.79) |
|  | Site 6 (vs. Site 1) | | -0.29 (0.35) | | 0.12 (0.79) |
|  | Site 7 (vs. Site 1) | | -0.27 (0.35) | | 0.10 (0.79) |
|  | Site 8 (vs. Site 1) | | -0.38 (4.83) | | 0.00 (4.57) |
|  | Site 9 (vs. Site 1) | | 0.07 (1.75) | | -0.02 (5.27) |
|  | Site 10 (vs. Site 1) | | -0.13 (0.32) | | 0.00 (0.79) |
|  | Site 11 (vs. Site 1) | | -0.21 (0.36) | | 0.15 (0.79) |
|  | Site 12 (vs. Site 1) | | -0.42 (0.35) | | 0.18 (0.79) |
|  | Site 13 (vs. Site 1) | | -0.25 (0.35) | | 0.07 (0.79) |
|  | Site 14 (vs. Site 1) | | -0.35 (0.35) | | -0.01 (0.79) |
|  | Site 15 (vs. Site 1) | | -0.31 (0.35) | | 0.05 (0.79) |
|  | Site 16 (vs. Site 1) | | -0.26 (0.34) | | -0.03 (0.79) |
|  | Site 17 (vs. Site 1) | | -0.49 (0.36) | | 0.14 (0.79) |
|  | Site 18 (vs. Site 1) | | -0.27 (0.34) | | 0.05 (0.79) |
|  | Site 19 (vs. Site 1) | | -0.64 (0.37) | | 0.03 (0.79) |
|  | Site 20 (vs. Site 1) | | -0.36 (0.34) | | 0.08 (0.79) |
|  | Site 21 (vs. Site 1) | | -0.25 (0.35) | | 0.08 (0.79) |
| Intercepts | |  | 0.18 (0.16) | | -0.27 (0.19) |
| Random effects | |  | 0.41 (0.01)*** | | 0.43 (0.01)*** |
| *Note*. * *p* < .05, ** *p* < .01, *** *p* < .001, after we used the Benjamini-Hochberg method to account for multiple testing. *CLSE = Criminal legal system exposure.* | | | | | |

**Figure S1**

*A Visual of Each Youth’s Trajectory of their Pubertal Development Score across Three Waves*

*Note*. For visual purposes, the trajectories were based on a 15%-randomly selected subset of youth in the analytic sample.
